# Advancing mRNA technologies for therapies and vaccines: An African context

**DOI:** 10.3389/fimmu.2022.1018961

**Published:** 2022-10-24

**Authors:** Dylan Kairuz, Nazia Samudh, Abdullah Ely, Patrick Arbuthnot, Kristie Bloom

**Affiliations:** Wits/SAMRC Antiviral Gene Therapy Research Unit, Faculty of Health Sciences, University of the Witwatersrand, Johannesburg, South Africa

**Keywords:** mRNA, saRNA, mRNA immunogenicity, mRNA modifications, mRNA vaccines, lipid nanoparticles, lyophilization, African vaccine development

## Abstract

Synthetic mRNA technologies represent a versatile platform that can be used to develop advanced drug products. The remarkable speed with which vaccine development programs designed and manufactured safe and effective COVID-19 vaccines has rekindled interest in mRNA technology, particularly for future pandemic preparedness. Although recent R&D has focused largely on advancing mRNA vaccines and large-scale manufacturing capabilities, the technology has been used to develop various immunotherapies, gene editing strategies, and protein replacement therapies. Within the mRNA technologies toolbox lie several platforms, design principles, and components that can be adapted to modulate immunogenicity, stability, *in situ* expression, and delivery. For example, incorporating modified nucleotides into conventional mRNA transcripts can reduce innate immune responses and improve *in situ* translation. Alternatively, self-amplifying RNA may enhance vaccine-mediated immunity by increasing antigen expression. This review will highlight recent advances in the field of synthetic mRNA therapies and vaccines, and discuss the ongoing global efforts aimed at reducing vaccine inequity by establishing mRNA manufacturing capacity within Africa and other low- and middle-income countries.

## 1 Introduction

Poor access, efficacy or complete lack of appropriate vaccines and therapies for infectious diseases, genetic disorders, and cancers, have highlighted the need for novel technologies to overcome these hurdles. Although messenger RNA (mRNA) was first investigated as a therapeutic in 1992 (1), complications including stability and immunogenicity limited the feasibility of the technology for further drug development, leading to the prioritization of DNA-based platforms (2). Recent advances in mRNA immune modulation, stabilization, purification, and delivery, has brought mRNA to the forefront of nucleic acid therapy development.

The rapid adoption of mRNA vaccines to combat the COVID-19 pandemic highlights the potential of this technology, particularly for pandemic preparedness, over that of more established technologies such as recombinant viruses. Recombinant viruses are very efficient at delivering vaccine and therapeutic sequences for expression within target cells and as a consequence their use is being actively explored in these fields (reviewed in (3, 4)). However, *in vitro* transcribed mRNA has several advantages over viral vectors such as recombinant adeno-associated viruses, adenoviruses and lentiviruses. An important consideration for clinical use is the immune response to the vector which limits re-administration. Once a viral vector is administered the host develops vector-associated immunity, thereby effectively preventing re-administration of the same serotype. *In vitro* transcribed mRNA is delivered using non-viral vectors which do not suffer from the same shortcoming. Another advantage of using mRNA technology over that of recombinant viral vectors lies in the relative ease of scaling up manufacturing. Since the mRNA is produced using cell-free processes, there is no requirement, as is the case for viral vector production, for producer cells. One disadvantage of mRNA technology lies in the inherent instability of RNA requiring storage of the drug product at temperatures as low as -80°C, which, for logistical purposes, becomes infeasible in low- and middle-income countries. Improvements in thermostability and lyophilization of formulations that allow storage at 4°C and 25°C are actively being explored to mitigate the need for ultralow freezers. The versatility and clinical relevance of mRNA technologies for pandemic preparedness has created a global demand for manufacturing of these vaccines and therapeutics.

There are two main types of mRNA technologies: conventional mRNA and self-amplifying mRNA (saRNA) (reviewed in (5)). The basic components of an RNA transcript include a 5’ cap and 5’ untranslated region (UTR), open reading frame (ORF), 3’ UTR, and polyadenosine (polyA) tail, all of which are essential for *in situ* protein translation. saRNAs require additional components in the form of sequences encoding four non-structural proteins (nsP1-4) derived from Alphavirus genomes which form the RNA-dependent RNA polymerase (RdRP) complex, and the 5’ and 3’ conserved sequence elements (CSEs), both of which are required for self-propagation of the RNA. The sequence encoding the gene of interest is typically included downstream of nsP1-4, under the control of a subgenomic promoter.

Synthetic mRNA (or saRNA) production starts with the design and synthesis of a DNA template which encodes the necessary components of the transcript downstream of a bacteriophage promoter (usually T7, T3 or SP6). The mRNA can then be transcribed from the linear template in a highly efficient and cell-free process known as *in vitro* transcription (IVT). In its simplest form, this requires an RNA polymerase corresponding to the promoter encoded in the template, ribonucleotide triphosphates (rNTPs), and appropriate buffer. Capping of the mRNA can be achieved during IVT with co-transcriptional cap analogs. Alternatively, enzymatic addition of a 5’ cap is possible. Purified mRNA is subsequently formulated in a lipid nanoparticle containing a mixture of cationic or ionizable lipids and excipients. Upon entry into the target cell, the protein is immediately translated using host translational machinery and can function within the cytoplasm, be trafficked to the cell-membrane or nucleus, or secreted, depending on the design of the mRNA. In the case of saRNA, the initial *in situ* translation of the nsP1-4 proteins ensures the exponential replication and subsequent translation of the subgenomic RNA. A major advantage of this platform is that *in situ* translation of mRNA in the cytoplasm of host cells allows for native protein folding and post-transcriptional modifications. As such, therapeutics and antigens are expressed in conformations that often mimic native host proteins or viral epitopes, respectively. Other key attributes, including reduced genotoxicity, flexible modular design principles, and small manufacturing footprints have helped mRNA platforms overcome limits of traditional vaccine production pipelines. Importantly, this platform may be considered a stepping-stone for disease-burdened developing countries to gain vaccine manufacturing independence.

## 2 Modification of mRNA to improve efficacy

**Figure 1:**
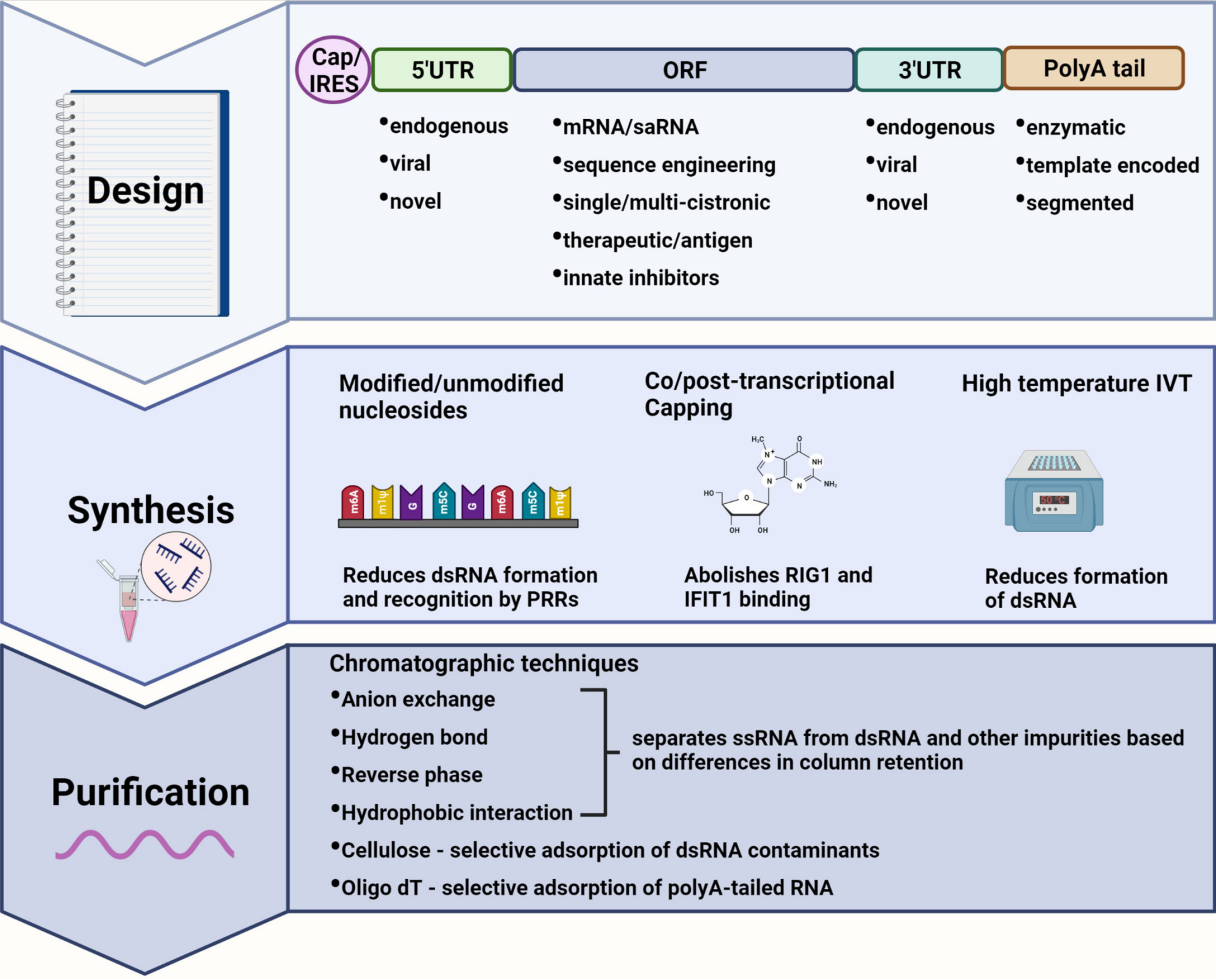
Immune modulating modifications to mRNA design, synthesis, and purification. **Design:** The mRNA transcript can be designed to incorporate elements of reduced immunogenic potential. A 5’ cap abolishes recognition by cytosolic pattern recognition receptors (PRRs) whereas an internal ribosome entry site (IRES) allows for cap-independent translation during interferon-induced translational shut-off. Untranslated regions (UTRs) from highly expressed endogenous genes, or alphavirus derived conserved sequence elements (CSEs) in the case of self-amplifying RNAs (saRNAs), ensure efficient translation of the mRNA. Highly structured viral CSEs are also capable of evading PRR recognition and binding. The sequence encoding therapeutic proteins or antigens can be codon optimized to deplete uridine thus reducing recognition by PRRs. A polyA tail enhances mRNA stability and translational efficiency with template encoded polyA tails inhibiting the formation of double-stranded (dsRNA) by-products. **Synthesis:** Modified nucleotides may be incorporated during *in vitro* transcription (IVT) to produce transcripts that evade PRR recognition while also reducing the formation of dsRNA. Capping can be performed co/post-transcriptionally to abolish binding by RIG-I and IFIT1. IVT reactions performed at higher temperatures also reduce the formation of dsRNA by-products. **Purification:** dsRNA as well as other impurities can be separated from single-stranded RNA (ssRNA) by a variety or combination of chromatography-based purification techniques enabling the recovery of pure mRNA product with reduced immunogenicity. Created using BioRender.com.

In the case of mRNA vaccines and therapies, uncapped or cap 0 ssRNA, uridine-rich ssRNA, and dsRNA are common well-characterized triggers of innate immunity. For mRNA-based therapeutics, it is important to mitigate the innate immune response to allow accumulation of the therapeutic and prevent immune-mediated clearance. For vaccine applications, induction of interferons and pro-inflammatory cytokines may improve adaptive immune responses but could also prematurely inhibit translation of the antigen which can result in sub-optimal adaptive immunity (6). mRNA stability is another major concern, as it is prone to hydrolysis and enzymatic degradation during synthesis, storage, and vaccine administration (7). Over the past few years, the collective contributions of many scientists have led to the improvement of synthetic mRNA production, such that the immunogenicity, stability, and translation of these transcripts can now be modulated to suit the application. These modifications can be implemented during the design, synthesis, and purification steps of mRNA production (**Figure 1**). A summary of the key modifications, their advantages and disadvantages, as well as some examples of mRNA therapies in clinical trials, are summarized in **Table 1**.

**Table 1:** Advantages and disadvantages of key modifications to *in vitro* transcribed mRNA, with selected examples of applications in clinical trials.

Modifications to mRNA	Advantages	Disadvantages	Applications in clinical trials	Refs.
**Sequence engineering with unmodified nucleotides**	Expensive modified nucleotides are not required Decreased native mRNA immunogenicity through uridine depletion Increased rate of translation	Requires careful optimization to avoid incorrect folding of the *in situ* translated protein which can lead to alterations in functionality or immunogenicity	SARS-CoV-2 (NCT05260437) Influenza (NCT05252338) Rabies (NCT03713086)	(8–11)
**Unmodified nucleotides**	Expensive modified nucleotides are not required Self-adjuvanting	Innate stimulation can have a negative impact on translation Not suitable for therapies that require prolonged protein expression	Melanoma TAA (NCT04526899) Prostate cancer TAA (NCT04382898) Personalized cancer vaccines (NCT04486378) Cystic fibrosis (NCT03375047; Phase I/II trials showed lack of efficacy)	(12)
**Modified nucleotides**	Increased stability and expression Reduced immunogenicity Suitable for therapeutic or vaccine applications requiring repeat doses	Additional manufacturing costs May destabilize functional secondary structures formed by IRES or saRNA	SARS-CoV-2 - Comirnaty, Spikevax (Approved http://www.fda.gov/) and numerous candidates in clinical trials (reviewed in (13)) Influenza (NCT05052697) HIV (NCT05001373) Zika (NCT04917861) Tuberculosis (NCT05537038) Chikungunya virus (NCT03829384) Anti-claudin 18.2 antibody (NCT04683939) Interleukin-2 (NCT04455620) Vascular endothelial growth factor A (NCT03370887) Propionic acidemia (NCT04159103) Methylmalonic acidemia (NCT04899310) Glycogen storage disease 1a (NCT05095727)	(14–16)
**Co-transcriptional capping (Cap1)**	Protected from 5’-3’ exonucleases Decreased native immunogenicity Streamlined production of capped mRNA High capping efficiency	Highly efficient cap analogs are expensive Relies on complementary base-pairing to initiating nucleotides on template	Widely used capping strategy e.g., Comirnaty	(17–19)
**Post-transcriptional enzymatic capping (Cap 1)**	Protected from 5’-3’ exonucleases Decreased native immunogenicity High capping efficiency that is not template sequence dependent	Longer manufacturing times Increased handling of mRNA may affect integrity and yield May not be ideal for longer saRNA transcripts which are more prone to degradation	Widely used capping strategy e.g., Spikevax	(17, 20)
**IRES**	Can express proteins without expensive cap analogs Does not require immune-dampening modifications since translation occurs in a cap-independent manner Multiple IRESs facilitate increased protein expression or expression of multiple proteins	Increased size of transcript Proteins in multicistronic constructs not always expressed in equimolar amounts IRES may inhibit the translation of certain proteins Not suitable for pandemic vaccine development because of increased optimization requirements May be more prone to exonuclease degradation	* None currently in clinical trials	(21–25)
**saRNA**	Expensive modified nucleotides are not required for manufacturing Effective at lower doses compared to non-replicating mRNA Self-adjuvanting Suitable for vaccine applications	Increased size of transcript Inherently immunogenic Therapeutic applications may require co-expression of innate inhibiting proteins	SARS-CoV-2 (NCT05435027) Rabies (NCT04062669) Influenza (NCT05227001) CEA antigen expressing solid tumors (NCT00529984) Colon cancer neoantigens (NCT05456165) HER2 Breast cancer (NCT03632941) HPV Cervical cancer (NCT03141463)	(19, 26, 27)

CEA, Carcinoembryonic antigen; HER2, Human Epidermal Growth Factor Receptor 2; HPV, Human Papilloma virus; IRES, internal ribosome entry site; mRNA, messenger RNA; saRNA, self-amplifying RNA; TAA, tumor-associated antigens.

### 2.1 mRNA and the innate immune system

**Figure 2:**
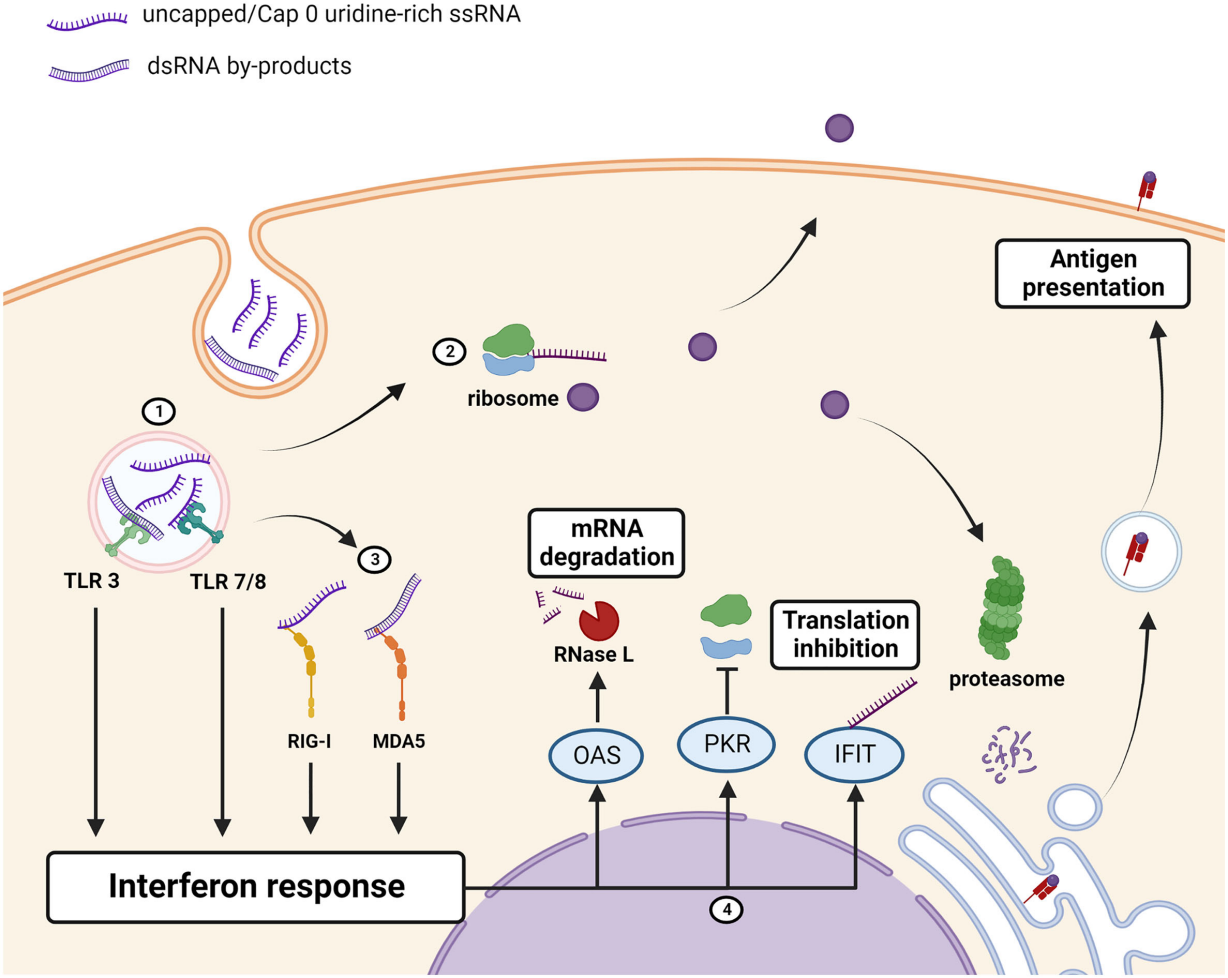
Immunogenicity of unmodified mRNA therapeutics/antigen. **(1)** Uridine-rich ssRNA and dsRNA IVT by-products, taken up by the cell through endocytosis, are sensed by toll-like receptors (TLRs) which induce an interferon response. **(2)** mRNA is translated into the protein of interest which can be retained intracellularly, displayed on the cell-membrane, or secreted. The protein of interest is also processed and bound to MHC for antigen presentation to the adaptive immune system. **(3)** Cytosolic PRRs RIG-I and MDA5 detect the presence of uncapped, uridine rich, or dsRNA and augments the interferon response. **(4)** Interferon-stimulated genes encoding antiviral proteins OAS, PKR and IFITs are upregulated. OAS activates RNase L which cleaves RNAs, PKR inhibits ribosome recruitment, and IFIT binds to uncapped or cap 0 mRNAs to prevent translation thus reducing the amount of the protein that is produced. Created using BioRender.com.

Understanding and tempering of the innate immune response to improve therapeutic efficacy has been a significant achievement in the field. The innate immune system is equipped with a variety of endosomal toll-like receptors (TLRs) and cytosolic pattern recognition receptors (PRRs) to sense pathogen-associated molecular patterns (PAMPs) that are characteristic of viral RNAs (reviewed in in (28)). Activation of these PRRs initiates a cascade of cellular pathways which culminates in secretion of type 1 interferons (interferons α and β) and pro-inflammatory cytokines (**Figure 2**). Type 1 interferons stimulate production of proteins that promote an antiviral state within the cell, whereas the concurrent release of pro-inflammatory cytokines creates an extracellular milieu that is conducive to the recruitment of immune cells and development of an appropriate adaptive immune response. PAMPs include polyuridine sequences, guanosine-uridine (GU) dinucleotide motifs, uncapped or cap 0 ssRNAs, or dsRNA viruses or replication intermediates of ssRNA viruses. TLR 7, found in plasmacytoid dendritic cells and B cells, stimulates secretion of interferon upon binding to guanosine and polyuridine sequences present on single-stranded viral RNAs (29). Myeloid dendritic cells and monocytes express TLR 8 which binds to uridine and GU motifs and promotes the secretion of pro-inflammatory cytokines (30). TLR 3 is more widely distributed and found on many innate immune cells as well as fibroblast and epithelial cells (reviewed in (28)). This PRR recognizes dsRNA and induces the secretion of IFN α, inflammatory cytokines and, when activated in dendritic cells, enhances antigen presentation.

Cytosolic PRRs are not restricted to cells of the innate immune system and are found in most cells. The cytosolic helicases, retinoic-acid inducible gene I product (RIG-I) and melanoma-associated differentiation antigen 5 (MDA5) induce interferon secretion following sensing of short and long (>2kbp) dsRNAs respectively (reviewed in (28)). RIG-I is also strongly activated by uncapped, and polyuridine-rich sequences present in ssRNAs. The secretion of interferon in response to activation of the above-mentioned PRRs promotes upregulation of antiviral proteins with PRR capabilities, such as 2’-5’-oligoadenylate synthetase (OAS), protein kinase R (PKR) and interferon-induced proteins with tetratricopeptide repeats (IFITs) [reviewed in (31, 32)]. OAS is activated by dsRNA and in turn activates RNase L which cleaves viral dsRNAs and provides substrates for RIG-I and MDA5. PKR, activated by RNA containing double-stranded regions and uridine, phosphorylates eukaryotic translation initiation factor 2 α (eIF2α) causing inhibition of translation (33). IFITs bind to eIF3 to prevent formation of the pre-initiation complex that is required for translation, and binds to mRNAs that lack a cap 1 moiety to prevent its translation (reviewed in (31)).

### 2.2 Design principles for conventional mRNA: Sequence engineering, UTRs, and tails

The most critical aspect of developing any mRNA-based therapeutic or vaccine is the design of the construct. Each region of the mRNA transcript has a role to play in modulating immunogenicity, stability and translatability of the transcript and thus should be chosen carefully to achieve the therapeutic objective. The mRNA transcript can be designed to encode recombinant therapeutic proteins, whole antigenic proteins, or multiple antigenic peptides. Based on the application, the amount of protein that is required can vary.

Sequence engineering approaches are used to enhance translation of the gene of interest. Thanks to the degeneracy of the genetic code, rare codons can be replaced by more abundant synonymous codons to increase the rate of translation which has also been shown to delay deadenylation of the polyA tail and mRNA decay (reviewed in (8) (9);). Sequence engineering can also be used to reduce the uridine composition of open reading frames (ORFs), especially those encoding pathogen-derived proteins, by using synonymous GC rich codons (10). This reduces TLR recognition while still encoding the same amino acid. This approach was successfully employed to produce high levels of erythropoietin in the absence of other modifications to the mRNA transcript (34).

However, codon optimization may not always be beneficial as altering codon usage and the rate of translation of certain proteins can affect folding, post-translational modifications, functioning, and antigenicity of the protein (reviewed in (11)). For mRNA therapies aimed at replacing endogenous proteins proper folding and post-translational modifications are essential to achieving the therapeutic objective. A more dire consequence of incorrect folding and post-translational modifications is inadvertent recognition of the therapeutic protein as an antigen and the development of an adaptive immune response. This unfortunate consequence was observed in patients that developed pure red cell aplasia following treatment with recombinant erythropoietin bearing altered glycosylation patterns (35). For whole antigen-encoding mRNA vaccines, preserving the conformation of epitopes is crucial to development of the appropriate protective adaptive response. Thus, codons need to be carefully optimized to suit the application while avoiding unintended consequences.

UTRs, located on either side of the ORF, are responsible for post-transcriptional regulation of genes. Mammalian 5’ UTRs vary greatly in length and secondary structure with longer, and more structurally stable UTRs correlating with reduced cap-dependent ribosome scanning and translation efficiency ( (36); reviewed in (21)). The 3’ UTR on the other hand contains elements such as microRNA (miRNA) binding sites and AU rich motifs that regulate mRNA stability and half-life (reviewed in (37)). A widely adopted approach to increasing translation and stability of synthetic mRNAs is to incorporate the UTRs of genes that are known to be highly expressed, since these UTRs are designed to ensure prolonged expression. The UTRs of human α- or β-globin have been favored in the design of many synthetic mRNAs. Serendipitously the head to tail arrangement of two β globin 3’ UTRs was shown to further enhance protein expression (38). An alternative to using globin-derived UTRs is to employ a systematic evolution of ligands by exponential enrichment (SELEX) approach to identify naturally occurring RNA segments that stabilize mRNAs more efficiently (39). Two such segments, derived from the human mitochondrial 12S rRNA (mtRNR1) and the 3’ UTR of mRNA encoding the human amino-terminal enhancer of split (AES) gene, outperformed globin 3’ UTRs when combined to form novel heterologous UTRs. Notably, these segments were predicted to contain fewer miRNA binding sites which contributed to the longevity of the mRNA transcripts in dendritic cells. Efficacy of these UTRs in other cell types may vary though as miRNAs are known to be differentially expressed between different cell types (reviewed in (40)). It is interesting to note that both the Pfizer/BioNTech and Moderna mRNA vaccines contain a combination of globin-derived and novel UTRs (reviewed in (41)). BNT162b2 is composed of the 5’ UTR of highly expressed α-globin and a modified AES-mtRNR1 3’ UTR. In contrast, Moderna chose to design a novel 5’ UTR while opting for the 3’ UTR of α-globin. Both these vaccines effectively elicit immune responses against the spike protein, albeit at differing doses.

Highly structured internal ribosome entry sites (IRESs), derived from the 5’ UTRs of some human and viral transcripts, can also be integrated into synthetic mRNAs to recruit the ribosome and initiate cap-independent translation (reviewed in (21)). A benefit of this transcript design for mRNA vaccines is that the immunogenicity of the mRNA does not need to be dampened and its self-adjuvanting properties can be exploited without concerns of insufficient antigen production. In addition, capping of IRES-containing mRNAs may be considered an unnecessary step and avoids high costs associated with capping. When compared to capped luciferase-encoding mRNAs, uncapped IRES-containing mRNAs produced significantly more luciferase, with expression peaking around 8 hours and decreasing to undetectable levels at day 4 post transfection (42). In the same study, dendritic cells transfected with either IRES or capped OVA-encoding mRNAs elicited comparable T-cell responses when introduced in mice and further protected them from tumor challenge. Alternatively, incorporation of an IRES may be used to produce multi-cistronic synthetic mRNA (22). The first gene is translated in a cap-dependent manner whereas downstream genes are translated by IRES-mediated ribosome recruitment. However, translation of the downstream gene in bi-cistronic constructs are often less efficient and certain upstream mRNA sequences, such as mRNA encoding Firefly luciferase (Fluc), can hinder IRES-dependent translation (23, 24). Thus, the arrangement of genes in multi-cistronic mRNAs needs to be optimized to ensure the efficiency of IRES-mediated translation initiation. Ko and colleagues tested the efficacy of various endogenous and viral IRES sequences and constructed an uncapped bi-cistronic mRNA that contained an unconventional polyA sequence at the 5’ end of the coxsackievirus B3 IRES, and the encephalomyocarditis virus IRES, from which *Renilla* and Firefly luciferases were simultaneously translated (25). The addition of the 5’ poly A tail was shown to increase the stability and translatability of the uncapped mRNA; however, this effect was specific to the coxsackivirus B3 IRES and the *in vivo* efficacy of such constructs needs to be explored further. It must be noted though that uncapped transcripts are susceptible to 5’-3’ exonuclease degradation, and thus may not be ideal for therapies requiring long-term protein expression. Nevertheless, this mRNA platform could be an attractive alternative for producing multivalent mRNA vaccines in developing countries where the cost of manufacturing modified capped mRNA may be inhibitory.

Incorporation of a polyA tail following the 3’ UTR is beneficial for cap-dependent translation (reviewed in (43)). This homopolymeric stretch of adenine nucleotides works in concert with polyA binding proteins, translation initiation factors and the 5’ cap to form a stable complex which then recruits the 40S ribosomal subunit thus enhancing translation along a ‘closed loop’ of mRNA. This interaction also protects the mRNA from exonucleases present in the cytoplasm. However, as part of the mRNA regulation process, polyA tails are subject to deadenylation which hastens 3’-5’ exonuclease-mediated decay of the transcript. This shortening of the polyA tail eventually also leads to decapping of the transcript which results in 5’-3’ exonucleolytic attack. To postpone degradation and increase the stability of synthetic mRNA, longer polyA tails of up to 120 adenines is preferable (38). The polyA tail can be added enzymatically following transcription, however this may result in transcripts of various lengths. To reduce production time and costs, and achieve transcripts of a uniform and defined length, it is preferable to encode the polyA tail within the template (38). The polyA tail can also be encoded as shorter segments separated by spacer regions to reduce the complications of recombination that occur in *E. coli* during plasmid propagation (44). As a further advantage, template encoded polyA tails have also been shown to reduce the formation of immunogenic anti-sense dsRNA contaminants that are produced during IVT (45).

### 2.3 Design principles of saRNA – CSEs and immune modulation

In contrast to the variety of UTRs that are available for use in conventional non-replicating mRNAs, saRNAs depend on structured alphaviral genomic and subgenomic 5’ UTRs, referred to as CSEs for RNA replication, translation, and innate immune system evasion (reviewed in (26)). The relatively short 5’ CSE forms a stem-loop structure downstream of a highly conserved initiating AU dinucleotide and both these motifs are necessary to recruit viral and host factors for RNA replication. The proximity of the stem-loop to the cap 0 structure is also responsible for shielding the mRNA from the binding and inhibitory effects of IFIT1. The 3’ UTR contains a conserved sequence element that is also necessary for replication. Polyuridine (ligand for TLR 7) and AU rich motifs are also a feature of alphaviral 3’ UTRs and these bind to Human antigen R (HuR) proteins which prevents deadenylation thereby increasing the stability and half-life of viral mRNA transcripts. The sequestration of HuR by alphaviral 3’ UTR elements can reduce the stability of host mRNAs, such as those encoding the Polo-like kinase 2 (PLK2) (46). The downstream effect of decreased PLK2 stability is reduced IFNβ secretion in the presence of RLR ligand binding. Thus, although saRNA UTRs are immunogenic they are also equipped with unique features to rescue translation and thus modifications to these regions should be analyzed carefully.

saRNAs are not amenable to most design and synthesis modifications used in conventional mRNA designs, therefore complete elimination of innate stimulation is not easily achievable. However, co-expression of innate inhibiting proteins can be used to dampen interferon responses. This approach, using vaccinia virus-derived immune evasion proteins E3, K3 and B18 proteins, effectively inhibited the interferon pathway and increased translation of saRNA *in vivo* (27). To ensure that innate inhibiting proteins co-localize with the therapeutic protein or antigen, the genes can be encoded within the same transcript, although in the case of saRNAs, this will increase manufacturing complications (47).

### 2.4 Advances in mRNA synthesis: modified nucleotides, capping, and purification

Endogenous eukaryotic RNAs undergo extensive post-transcriptional modifications (PTMs). A key function of eukaryotic PTMs is to assist the innate immune system to distinguish between self and non-self (reviewed in (48)). IVT mRNAs synthesized using canonical nucleotides do not undergo these modifications and stimulate the innate immune system by activation of endosomal TLRs (12). Taking a cue from nature, Kariko et al. produced nucleotide-modified synthetic mRNAs which demonstrated increased stability and translatability, and most importantly, reduced immunogenicity (14, 15).

Modified nucleotides alter the physicochemical characteristics of native eukaryotic mRNA which in turn affects PRR recognition. N6-methyladenosine (m6A), a natural modification of adenosine, is known to reduce the thermodynamic stability of dsRNA structures (49). Thus, m6A modified mRNA can minimize recognition by dsRNA sensing PRRs such as TLR 3 and RIG-I (14, 50). In contrast, pseudouridine (Ψ) promotes base-stacking and stabilizes RNA duplexes, explaining why it is not as efficient at evading TLR 3 recognition (14, 51). Replacement of uridine motifs also significantly reduces recognition by the ssRNA sensing TLRs 7 and 8. mRNA containing Ψ does bind RIG-I with high affinity but is unable to induce the conformational changes that leads to an interferon response (50). The altered structure and increased stability of Ψ-modified mRNA also reduces OAS activation and hinders degradation by RNase L (33).

Further reductions in innate immune signaling can be achieved by using N1-methylpseudouridine (m1Ψ) and is extremely beneficial for mRNA-based therapeutics such as protein replacement therapies. This naturally occurring derivative of Ψ demonstrates even less TLR 3 signaling while enhancing translatability by increasing ribosome loading (52, 53). Another advantage is its inability to wobble-base pair and thus achieve translational fidelity [reviewed in (16)]. It is not surprising that m1Ψ has been favored for the development of mRNA vaccines against Zika, Influenza, HIV-1 (Human Immunodeficiency Virus), and Ebola, and more recently, against SARS-CoV-2 [reviewed in (16)].

While nucleotide modified mRNA vaccines have demonstrated a clear advantage over similar unmodified mRNA vaccines, it cannot be assumed that the same efficacy can be achieved for other constructs [reviewed in (16)]. Modifications that destabilize dsRNA may not be suitable for constructs that rely on stable secondary structures for translation (IRES), or replication and immune evasion (saRNAs). Stabilizing modifications have also been shown to produce secondary structures that are quite different from unmodified transcripts (54). The complete substitution of uridine with modified uridine will affect the transcript not only in the ORF but in the UTRs as well. Thus, the compatibility of modified nucleotides with each element of the mRNA transcript needs to be evaluated.

Eukaryotic mRNAs are co-transcriptionally capped at the 5’ end with an N7-methylguanosine (m7G/cap 0) which is linked in reverse to the first nucleotide by a 5’-5’ triphosphate bridge (reviewed in (17)). This modification is essential for cap-mediated translation and protection from 5’-3’ exonucleases. Uncapped mRNA is also immunogenic and is recognized by RIG-I and IFIT1 (55, 56). The cap 0 structure does reduce RIG-I recognition but can still induce an interferon response when present in high concentrations (55). IFIT1 PRRs also bind to cap 0 structures, sequestering them from the translational machinery (57). A further modification, in the form of 2’-O methylation of the first ribose, generates the cap 1 structure which functions to abolish recognition of endogenous mRNAs by RIG-I and IFIT1 (55, 57).

Capping can be performed enzymatically using the vaccinia virus capping system to generate cap 0 or cap 1 transcripts at close to 100% efficiency (58, 59). This system is not sequence specific and is highly suited for flexible industrial scale preparation of both vaccines and therapies (60). However, it adds an additional step in the mRNA manufacturing process. These hurdles can be overcome by co-transcriptional cap analogs, such as the popular CleanCap^®^ trinucleotides which incorporate natural cap 1 structures (10). The various CleanCap^®^ analogs function by complementary base-pairing to the first two initiating nucleotides following the T7 promoter sequence, a factor that needs to be taken into consideration during initial template design (10, 18).

#### 2.4.1 Reducing dsRNA by-products

The conventional process of IVT is highly prone to the formation of dsRNA by-products which serve as ligands to dsRNA-sensing PRRs, however, it is possible to avoid the formation of these immunogenic by-products. Anti-sense RNA can be eliminated or reduced by using modified nucleotides, such as Ψ, m1Ψ or 5-methylcytosine (m5C) (61). To prevent the formation of 3’ extension dsRNA by-products during IVT, a thermostable T7 RNA polymerase which functions optimally at 50°C can be used (45). This convenient adaptation to the IVT protocol, when used in combination with polyA-encoding templates, has been shown to be just as effective at reducing the interferon response as compared to purification using high performance liquid chromatography (HPLC). It remains to be seen if this adaptation is compatible with co-transcriptional cap analogs.

Post-transcriptional purification of synthetic mRNAs is necessary to remove immunogenic dsDNA templates and dsRNA by-products, aborted transcripts, excess reagents, and other chemical by-products to obtain full-length mRNA transcripts which would then be acceptable as pharmaceutical grade therapeutics and vaccines (reviewed in (62)). Removal of pDNA is simply achieved by DNase treatment, whereas various chromatographic techniques are available to remove the remaining contaminants. The presence of a polyA tail makes mRNA highly amenable to purification by affinity chromatography using oligo dT columns (63). Anion-exchange, hydrogen bond, hydrophobic interaction and ion-pair reverse phase chromatographic techniques are also efficient at binding ssRNA while eluting dsRNA and other contaminants under specific buffer conditions (64–66). Cellulose-based chromatography, on the other hand, specifically binds to and removes dsRNA while eluting ssRNA, however this has only been achieved on a small scale (67). Both ion-pair reverse phase chromatography and cellulose chromatography are similarly efficient at removing most contaminants resulting in elimination of the residual interferon response, and improved protein translation (66, 67). Cellulose-based purification can be performed in a shorter timeframe, excludes the use of harmful solvents, is more cost-effective, and allows for a greater recovery of full-length mRNA at a milligram scale (67). The yield of longer saRNAs or mRNAs with regions of dsRNA may however be negatively impacted since these regions will bind to the cellulose (65). Choice of purification method would thus come down to cost and ease of use.

## 3 Delivery of RNA therapeutics and vaccines using non-viral vectors

**Figure 3:**
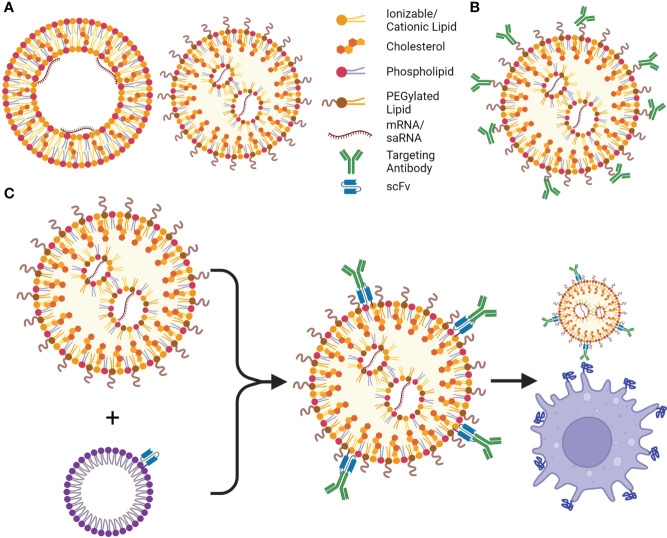
LNPs and their targeting advancements. **(A)** Cationic lipid nanoparticles (cLNPs) (left) and ionizable cationic LNPs (iLNPs) (right), are the most utilized non-viral delivery systems for current mRNA vaccines and therapeutics, cLNPs are composed of a lipid bilayer with an aqueous core where the mRNA interacts with the cationic lipids, cLNPs can be targeted to the spleen or lungs based on their charge. iLNPs consist of an electron-dense core, and are intrinsically targeted to the liver, however cationic lipids can be introduced to alter targeting to other organs (SORT, Selective Organ Targeting). **(B)** Antibodies or other targeting moieties can be conjugated to PEG or cholesterol to be targeted to different cell types; however, their orientation may prevent presentation of the correct epitope or binding site, therefore more of the moiety must be added to the LNP. **(C)** ASSET (anchored secondary scFv enabling targeting) LNPs solve this shortcoming by incorporating an scFv to the LNP, allowing for potentially any cell type to be targeted by adding a rat antibody targeting a surface molecule or receptor to the LNPs. This is a simple process using *E. coli* to produce the lipidated anti-rat scFv, which is incorporated into cholesterol micelles, and can be added to the LNPs followed by addition of the desired antibody. Created using BioRender.com.

Although mRNA is an extremely modular platform with high potential for future pharmaceutical drug development, they require lipid- and/or protein-based delivery systems to enter the cell cytoplasm and confer protection against RNase degradation. Lipid-based systems were first discovered in the 1960s by Bangham, when liposomes, or cationic lipid nanoparticles (cLNPs), were observed to spontaneously form vesicles in aqueous solutions (68, 69). Thirty years later, Doxil^®^, a liposomal-formulated doxorubicin, was approved in the United States. Since then, multiple liposome (reviewed in (70)) and LNP (containing ionizable cationic lipids, iLNPs) drugs have been approved for clinical use by the FDA (71, 72). LNPs usually comprise four components: a cationic lipid, a phospholipid, cholesterol, and a PEGylated lipid (reviewed in (73)). iLNPs consist of an ‘electron dense’ structure (**Figure 3A**) and contain a lipid that is cationic at an acidic pH, allowing for RNA complexation and endosomal release, but remains neutral at physiological pH (74). These are widely used to deliver RNA therapeutics to liver cells as they have a natural tropism mediated by surface adsorption of serum ApoE which facilitates low density lipoprotein receptor (LDLR) binding and subsequent transfection (75). Ionizable lipids have been widely used to deliver siRNA (small interfering RNA), mRNA and saRNA as therapies and vaccines, with both the Pfizer/BioNTech and Moderna COVID-19 vaccines, and Onpattro (an siRNA therapeutic) making use of an ionizable lipid (76–78). However, iLNPs would benefit from further optimization to improve targeted delivery (79) and reduce the likelihood of unwanted side effects such as anaphylaxis (80) and myocarditis (81). cLNPs differ from iLNPs by making use of a permanently cationic lipids that form a lipid bilayer structure (**Figure 3A**) (82). Although historically cLNPs have not been the preferred choice for the delivery of RNA therapeutics, owing to rapid clearance after systemic injection, reports of toxicity *in vitro*, and unintended immune stimulation (83), they could be harnessed for mRNA and saRNA vaccine development (84). Their permanent cationic charge makes them highly effective in complexing with RNA. Other delivery systems including polymers such as Polyethyleneimine (PEI) (85) and pABOL (86) have also been investigated although their clinical value remains to be seen. Recently, Zhang and colleagues described ionizable dendrimers as single component nanoparticles, bypassing the need for cholesterol, phospholipids, and a PEGylated lipid (87). Dendrimers are uniform molecules consisting of a core which is surrounded by highly branched sidechains terminating in functional groups (88). “Janus” dendrimers, which these ionizable dendrimers are based on, consist of reacting two or more dendrons (sets of dendrimer branches or “wedges”) together, forming molecules with multiple properties. In this case, amphiphilic molecules were synthesized, allowing for nanoparticles to self-assemble (89). These nanoparticles can be engineered to mimic components of cell membranes by conjugating sugars to their hydrophobic region, and this, along with their ionizable properties and versatility of potential structure, make them excellent candidates for efficient mRNA delivery (90). However, development of biodegradable dendrimers are important to reduce toxicity of the platform (91).

### 3.1 Advances in targeted delivery of RNA therapeutics using lipid nanoparticles

Cell-specific targeting of mRNA therapies and vaccines may be crucial to developing safe and effective drug products. Conjugation of a cell targeting moiety (ligand or antibody) to the cholesterol or PEGylated lipid (**Figure 3B**) is commonly used to generate cell specific LNPs (75). For example, mannose has been used to target liver sinusoidal endothelial cells (LSECs) in the liver after intravenous injection (92) as well as antigen presenting cells in saRNA vaccines after intramuscular injection (93). Antibody conjugation has recently shown promise for CD4+ T cell targeting (94) albeit with leaky liver expression. The same group targeted lung and inflamed brain endothelial cells using anti-PECAM-1 (95) and -VCAM (96) antibodies conjugated to iLNPs and cLNPs respectively. However, conjugating antibodies to LNPs is inefficient and antibody-dependent optimization is required. Kedmi and colleagues overcame this issue by designing a modular platform, referred to as anchored secondary scFv enabling targeting or ASSET, which can incorporate any antibody onto the surface of an LNP (97). This method uses a plasmid encoding an N-terminal signal peptide, followed by a peptide motif resulting in lipidation by *E. coli*, a single chain variable fragment (scFv) targeting rat IgG, and a His-tag for purification. After purification, cholesterol is added to produce micelles, and lipidation allows incorporation of the scFv into the micelles, which are mixed with pre-formulated mRNA-LNPs to form ASSET-LNPs (**Figure 3C**). Potentially any rat IgG antibody can be added to these ASSET-LNPs to create targeted LNPs. ASSET also uses significantly less antibodies as each antibody is positioned in the correct orientation as a result of the scFv, unlike conventional conjugation methods (**Figure 3C** compared to 3B). The authors were able to target a plethora of cell markers including CD44, CD34, CD3, CD4, CD25, CD29, Ly6C and Itgb7, and treat models of inflammatory bowel syndrome (IBS) and disseminated bone marrow mantle cell lymphoma by targeting siRNA to Ly6C and CD29, respectively. RNAi was also “dual targeted” using ASSET-LNPs targeting PD-L1 to sensitize cancer cells to chemotherapy, while immune-boosting myeloid cells by knockdown of heme oxigenase-1 (98). This approach was further adapted to deliver CRISPR mRNA and sgRNA, improving gene editing and survival compared to an IgG isotype LNP in an aggressive orthotopic glioblastoma model (99). It could also deliver mRNA encoding therapeutic interleukin 10 (IL10) to treat IBS (100), again using anti-Ly6C antibodies with reduced toxicity and immunogenicity. In Fluc biodistribution studies, the same group showed a 10-fold reduction in non-specific liver targeting coupled with a 100-fold increase in expression in Ly6C+ cells.

**Figure 4:**
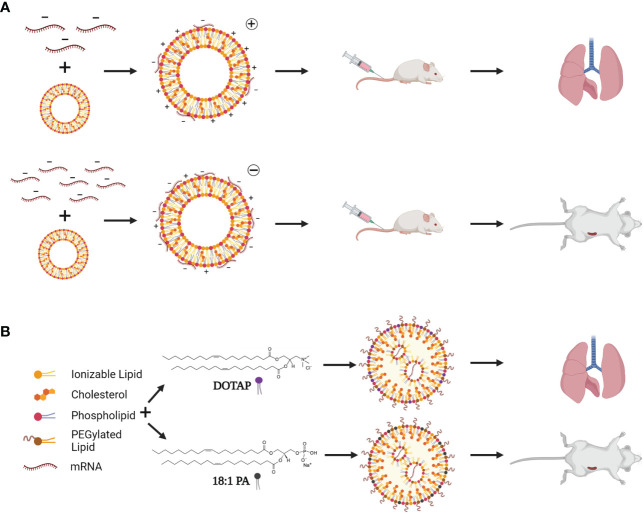
LNP targeting by charge switching and lipid choice. **(A)** By adding less mRNA (top), cLNPs are more positively charged, and target the liver after systemic injection. Addition of more mRNA to the exterior of cLNPs (bottom) changes the overall charge to negative, resulting in spleen targeting of cLNPs after systemic injection. **(B)** SORT (Selective Organ Targeting)-LNPs involve incorporating other targeting lipids such as DOTAP (1,2-dioleoyl-3-trimethylammonium-propane) and 18:1 PA (1,2-dioleoyl-sn-glycero-3-phosphate) to target specific organs depending on the lipid used and percentage of the lipid included. A high percentage of DOTAP allows for lung targeting, while lower percentages would target the spleen. Addition of 18:1 PA results in spleen targeting after systemic injection. Created using BioRender.com.

Biodistribution patterns of cLNPs on the other hand, can be altered by simply changing the charge of the LNP. Kranz and colleagues evaluated a range of N:P ratios (ratio of positively charged nitrogen on the lipid to negatively charged phosphate of the RNA), where a higher N:P (5:1) has less RNA, and a lower N:P (1:5) has more RNA (101). Higher N:P ratios were able to target the lungs, while lower N:P ratios targeted the spleen (**Figure 4A**). This technology was used to create a non-inflammatory mRNA vaccine to treat autoimmune encephalomyelitis using a low N:P ratio (102). This phenomenon was further explored by adding different percentages of various lipid components to a degradable dendrimer ionizable LNP, in a process referred to as Selective Organ Targeting (SORT; **Figure 4B**) (103). For example, adding lower and higher percentages of 1,2-dioleoyl-3-trimethylammonium-propane (DOTAP) improved lung and spleen delivery respectively, while 1,2-dioleoyl-sn-glycero-3-phosphate (18PA) directed spleen delivery (103). SORT was subsequently used for CRISPR-Cas9 delivery (mRNA and sgRNA) demonstrating its potential for the development of gene therapies. SORT was also applied in a study that designed multi-tailed ionizable phospholipids (iPhos) to improve RNA delivery (104). LNPs utilizing ionizable lipids such as Dlin-DMA-MC3 only allow release of 1-4% of the encapsulated RNA due to lack of protonation of inaccessible portions of the lipid, and hence, reduced endosomal escape (105–108). Impressively, the authors performed in-depth investigations into the cellular pharmacodynamics of these iPhos to optimize mRNA cargo release, including phase transition, endosomal membrane fusion, and membrane disruption. This, highlights important shortcomings of current LNP technology designs. Although the technology has been widely used, mRNA delivery can be optimized, and iterative design of lipids based on these essential factors could improve LNP functionality.

## 4 Application of mRNA technologies

With such a versatile platform, mRNA technologies have already been adapted into a plethora of therapeutics and vaccines including gene editing tools, protein replacement therapies, immunomodulatory drugs, and vaccines against infectious diseases and cancers.

As protein replacement therapies, mRNA is a viable candidate for the treatment of monogenic disorders, as native protein folding, and modifications will occur. mRNA encoding human factor IX was used to treat hemophilia B with a longer therapeutic effect than recombinant protein therapy, with the potential to further improve clotting activity (109). mRNA-based protein replacement therapies have also shown promise in numerous lung (110) and heart (111), and liver (112) diseases. Recently, Arcturus Therapeutics investigated a novel enzyme replacement therapy to treat phenylketonuria. mRNA encoding phenylalanine hydroxylase (PAH), which metabolizes phenylalanine (Phe) was delivered using their proprietary LUNAR LNPs. The authors combined UTR choice and gene of interest design to develop the optimal PAH-expressing mRNA, and LNP choice changed therapy efficacy as reduced rate of clearance improved Phe reduction after treatment (113). The LUNAR platform has also been applied to treat ornithine transcarbamylase deficiency (114) with clinical trials underway (NCT04442347). Similarly, mRNA-based enzyme replacement therapy for glycogen storage disease type 1a and type III are currently undergoing clinical evaluation by Moderna (NCT05095727) and Ultragenyx Pharmaceutical Inc (NCT04990388) respectively. Although recent modifications and delivery platforms have improved the stability, translatability and longevity of mRNA, these improvements may not be sufficient to sustain long-term expression of therapeutic proteins to treat genetic disorders which will undoubtedly require repeat mRNA injections. Substantially longer periods of expression (up to 60 days) can be achieved using saRNAs which, with the co-expression of innate inhibitors, will allow for a reduction in dosing frequency (62, 115). Fortunately, the transient nature of the mRNA platform can be leveraged to correct genetic disorders instead of regularly replacing the dysfunctional or deficient protein.

Designer nucleases including Zinc finger nucleases (ZFNs), Transcription activator-like effector nucleases (TALENs) and CRISPR (clustered regularly interspaced short palindromic repeats)-Cas9, have all been adapted to mRNA platforms allowing gene editing *in vivo* and *ex vivo* (116). Unlike DNA, the short half-life of mRNA reduces off-target effects, while maintaining sufficient expression length and longevity to allow for effective editing, especially as a result of recent modifications that reduce mRNA immunogenicity and improve stability. Recently, mRNA encoding Cas9 was delivered using novel LNPs, with single guide RNAs delivered in separate LNPs to treat Duchenne muscular dystrophy (DMD). A dose-dependent response was observed after direct injection into a muscle vein and by limiting delivery to the muscle, dystrophin was restored. This study also showed that multiple doses could be administered without sustained immune activation or inducing anti-Cas9 antibody titers (117). mRNA holds advantages over Cas9 protein delivery as foreign proteins could potentially result in immune activation (118).

Antigen vaccines (tumor associated or neoantigen), CAR (chimeric antigen receptor)-T cells and antibody therapies (119) have all shown potential as mRNA-based cancer therapies and vaccines (120). Antigen vaccines are highly explored immunotherapies, with multiple ongoing clinical trials (120, 121). Due to immunosuppression in cancer patients, delivery of mRNA with adjuvants (122, 123), checkpoint inhibitors (101) and cytokines [reviewed in (121)] have proven beneficial. The addition of mRNAs encoding co-stimulatory receptors and/or ligands has been shown to improve dendritic cell (DC) vaccines [reviewed in (121)]. Using mRNA to transiently express CARs aids in preventing viral integration of genes, which occurs when using lentiviral vectors, while simplifying production (reviewed in (120) (124, 125);). Many of these immunotherapies may be further improved when coupled with cell-targeted delivery based on charge switching cationic LNPs (cLNPs (101),), lipid-polymers nanoparticles (126), SORT LNPs and ASSET LNPs.

Conventional mRNA and saRNA have also been extensively investigated for the prevention of infectious diseases including SARS-CoV-2, Zika virus, cytomegalovirus, respiratory syncytial virus (RSV) and human immunodeficiency virus (HIV) (discussed further in section 4.1) (reviewed in (127)). While the majority of clinical trials have focused on conventional mRNA candidate vaccines, recent first-in human SARS-CoV-2 and Rabies saRNA vaccine studies will prove useful in determining the clinical potential this platform (5, 7). Its ability to self-replicate and ultimately improve antigen expression could lead to saRNA vaccines requiring lower doses than conventional mRNA. In a pre-clinical SARS-CoV-2 study, Blakney and colleagues showed that iLNP delivery of as low as 0.01 µg saRNA could induce a neutralizing antibody response after a single dose, and 0.1 µg saRNA could produce strong SARS-CoV-2 specific IgG and neutralizing antibodies, as well as cellular immune responses (86). Although Arcturus Therapeutic’s phase III COVID saRNA vaccine clinical trial only showed 55% efficacy (95% efficacy for preventing severe disease and death), this is possibly attributable to new circulating variants (128). Passive immunization has also been explored in the form of mRNA transcripts expressing HIV (129), RSV (130), and recently hepatitis B virus (HBV) (131) antibodies. In the case of RSV, antibodies were targeted to the lungs by nebulization. saRNA may be a more promising platform for passive immunization owing to longevity of protein expression, allowing for lower dosage and prolonged protection, as shown with Zika virus infections (132).

mRNA vaccines can also be used to prevent undesirable immune responses such as type 1 allergies and treating autoimmune diseases. Negatively charged cLNPs targeting the spleen were used to create a non-inflammatory modified mRNA vaccine to treat autoimmune encephalomyelitis using a low N:P ratio (102). This allowed presentation of autoantigen by antigen presenting cells with low co-stimulatory molecule expression, mimicking natural tolerance, which could treat a mouse model of multiple sclerosis. Type 1 allergies inducing type 2 helper CD4+ T cells can be combated by vaccines inducing a Th1 CD4+ T cell response, however an IgE response must be avoided [reviewed in (133)]. This involves inducing IgG2a antibodies blocking interactions of allergen with IgE antibodies, with interferon-gamma (IFN-γ) secretion by CD8+ T cells being important for the anti-inflammatory response. A panel of 29 mRNAs/saRNAs encoding different allergens resulted in a Th1-biased response with increased related cytokines (IFN-γ) and reduced or lack of antigen-specific IgE after sensitization (134). Later, this was shown to allow for long-term prevention of IgE release and Th1 memory response with related cytokines (135). However, naked mRNA was utilized in these studies, and immune response and dosage could be improved by formulation of mRNA in non-viral vectors.

### 4.1 Opportunities for utilizing mRNA vaccines against diseases burdening Africa

#### 4.1.1 Infectious diseases

African countries are burdened by a high prevalence of non-vaccine preventable infectious diseases such as malaria, HIV-1, and tuberculosis (TB). This disease burden is likely to rise as the true impact of local and international lockdowns aimed at preventing the spread of SARS-CoV-2 have negatively impacted other pillars of healthcare such as childhood immunizations, TB and HIV treatment regimes, and infectious disease screening and management.

##### 4.1.1.1 Tuberculosis

TB, caused by *Mycobacterium tuberculosis* (*Mtb*) infection, is a major cause of morbidity and mortality in South Africa, with 360 000 new infections in 2019 and 58 000 of those infections resulting in death (136). Although South Africa is reaching its recent “End TB Strategy” goals, challenges such as multidrug-resistant and extensively drug-resistant tuberculosis (128) may interfere with reaching these goals in the future. The currently approved TB vaccine, BCG (bacillus Calmette–Guérin), is only protective in children [reviewed in (137)], emphasizing the need to develop a broadly effective vaccine candidate for Africa. However, developing a vaccine candidate for TB is challenging, due to its inhibition of the innate immune response and variability in different populations [reviewed in (137)]. One study showed that, while the BCG vaccine conferred relatively good protection in children from the United Kingdom, there was no protection against disease in children from Malawi (138), possibly because of exposure of Malawian children to non-tuberculosis mycobacteria (139). Similarly, in South Africans, peripheral lipid-specific T cell responses did not correlate with *Mtb* infection, possibly due to continuous exposure to *Mtb* and other mycobacteria, BCG vaccination, or due to differential location of these T cells in the body compared to other populations (140). B and T cell frequencies are heterogenous between populations, and even within South Africans, broad epitope-specific responses are present (141, 142). *Mtb* response is also altered by HIV infection (143–145), which have a strong co-infection prevalence in South Africa (146). These factors, alongside studies on population specific cytokine responses will improve vaccine studies and design by predetermining the desired induced cytokine response (143). This highlights the importance of keeping the population of interest in mind during vaccine design and testing. Although there are many candidates in clinical trials, with a few in the phase II/III or phase III trials, efficacy is varied. mRNA was first used for a TB vaccine in mice in 2004, with protection less than that of BCG (147). Recently however, BioNTech have announced their plan to initiate clinical trials for their TB vaccine this year (148). A design for a TB mRNA vaccine has also been developed using *in silico* predictive programs (149). This used *Mtb* proteins that modulate host immune responses through epigenetic changes. These epigenetic changes include DNA methylation and Histone acetylation, which act to interfere with anti-inflammatory gene expression, such as overexpression of interleukin 10 (150). Specific peptides were chosen based on antigenicity, absence of auto-inflammatory and allergenic induction, and prediction of cytotoxic (CTL) and helper lymphocyte epitopes. Lymphocyte epitopes were also investigated for HLA binding, with one epitope binding 32 MHC II alleles (albeit some only binding one MHC allele). The final candidate included multiple (thirty) epitopes, a TLR 4 agonist, tissue plasminogen activator signal sequence and an MHC I trafficking signal (MITD). *In silico* analysis of an immune response after injection showed the potential of this vaccine to induce antibody production, memory B cell and subsequent cytolytic and helper T cell responses, although the actual practicality of this approach will require validation *in vivo*. Even though a population tool predicted 99.38% of the world’s population would respond to this vaccine based on HLA alleles, epitopes may not be suitable to Africa due to genetic diversity and HLA heterogeneity, both of which have undergone limited research (151, 152).

##### 4.1.1.2 HIV

Development of an effective HIV vaccine would have an extraordinary global impact with 37.9 million people living with HIV, 25.7 of which are in Africa, and 1.1 million new infections reported in 2018 (153). Despite years of research, conventional and next-generation vaccine approaches have yet to achieve prophylactic protection or therapeutic effect (154, 155). Recent mRNA-based candidates in clinical trials, including those developed by BioNTech (148), Moderna (NCT05001373) and the National Institute of Health (NIH) (NCT05217641), will test the applicability of the recently approved platform in diseases which have historically struggled to produce encouraging results. However, due to the genetic diversity of HIV and circulating recombinant forms (CRFs) dominating certain geographical regions, careful antigen selection may be needed to design effective vaccines for different regions (reviewed in (156)). The error-prone reverse transcriptase induces rapid formation of variants and so, vaccine candidates need to prevent infection and subsequent replication before these variants can form [reviewed in (157)]. Glycosylation of the glycoprotein also prevents antibody binding to antigenic epitopes (158), further complicating vaccine designs.

Although ongoing clinical trials are investigating vaccines that do not induce broadly neutralizing antibodies (bNAbs; NCT03060629, NCT03964415), a more accepted approach is to use an antigen that would induce bNAbs that neutralize multiple variants of HIV. For example, many designs make use of eOD-GT8 60-mer (germline targeting engineered outer domains) to induce VRC01-class bNAbs by targeting the CD4 binding site (159). This makes use of lumazine synthase, a bacterial protein, to induce formation of icosahedral nanoparticles. Although this design is used in the Moderna mRNA HIV vaccine and a pre-clinical trial for an HIV saRNA vaccine (160), it may be limited by non-bNAb recognition and reduce effectiveness of the vaccine (161–163). Duan et al. were able to improve the proportion of CD4 binding site-specific antibodies and B cells in mice by introducing NxT sequons (amino acid consensus sequences allowing glycosylation) into eOD-GT8, resulting in N-linked glycosylation of potential off-target binding sites and reducing priming of non-CD4 binding antibodies (164). These types of modifications could be adapted to mRNA technologies and other antigen designs to improve targeting, vaccine efficacy, and adaptability to different strains. Other vaccine designs make use of envelope trimers to mimic the natural HIV envelope, such as those recently initiated in the NIH clinical trial. However additional strategies may be required to maintain correct folding of the mRNA-encoded trimers (reviewed in (157)). Nevertheless, results from these mRNA clinical trials will help inform future HIV vaccine designs.

##### 4.1.1.3 Malaria

Malaria, caused by the protozoan parasite *Plasmodium*, affects a staggering number of people globally (241 million in 2020) and is life-threatening especially to children under the age of five and pregnant women (165). The deadliest of the *Plasmodium* species, *P. falciparum*, is endemic to Africa where approximately 95% of global malaria cases as well as deaths occurred in 2020. The complexity of the parasite’s life cycle, the polymorphic nature of its antigens, and its ability to modulate the host’s immune response has made it difficult to produce an efficient malaria vaccine [reviewed in (166, 167)]. This is evidenced by the sheer number of vaccine candidates that have failed to induce sufficient protective or sterilizing immunity. After almost three decades of arduous research, iterative development, and numerous clinical trials, RTS, S (GlaxoSmithKline), a recombinant virus-like particle vaccine based on the immunodominant circumsporozoite protein (CSP), became the first vaccine against malaria to obtain licensure (168). However, RTS,S is modestly immunogenic, reducing the instances of clinical malaria by only 26% in infants and by 36% in older groups (169). In addition, antibody titers wane after a short period demonstrating the need for vaccines that elicit more robust and durable protection.

Virtually all vaccine platforms have been explored for the development of a malaria vaccine, each with its own unique production and logistical challenges. Disappointingly, all these vaccines have failed dismally when applied in the field, possibly because these vaccines target single strains of *P. falciparum* while the recipients reside in areas where any number of heterologous strains may be circulating at any given time (170). For example, RTS,S is based on the NF54/3D7 strain, thought to be of African origin, and showed promising results in early trials. However, this strain is a poor representation of the haplotypes present in different regions of Africa, which may affect vaccine efficacy (170). It may be more beneficial to develop vaccines against the dominant strains within geographic regions, which will be costly and laborious, however, the result would be a vaccine that is tailored to the needs of the residing population. In addition, natural immunity, as observed in endemic areas, is acquired over many years of repeated and sustained exposure to many different strains of the parasite (171). This immunity, although not sterilizing, offers protection against the development of clinical malaria and hints at the need for multi-epitope vaccines that elicit strain-transcending immunity. With regards to ease of production and the cost-effectiveness of such vaccines, the modular mRNA or saRNA platforms may hold the advantage over other vaccine manufacturing platforms.

Recently, Mallory and colleagues developed a modified nucleoside mRNA-LNP vaccine targeting the CSP (3D7 strain) of *P. falciparum* (172). Two doses of the vaccine administered to mice elicited a balanced humoral and cytotoxic response, with a third dose enhancing the Th1 response. This type of immune response is necessary to prevent hepatocyte infection and eliminate infected hepatocytes should the humoral response fail. The vaccine also conferred sterilizing immunity to sporozoite challenge. In a novel approach, researchers at Yale University developed a saRNA vaccine targeting the conserved immune modulating *Plasmodium* macrophage migration inhibitory factor (PMIF) which is produced during the blood-stage of infection and suppresses the development an effective adaptive immune response (173). This vaccine inhibited PMIF and, more impressively, promoted the development of liver and blood stage antibodies and CSP-targeting liver-resident CD8+ T cells which enabled better immune control in infected mice, and protected cured mice from re-challenge. This study demonstrated how careful selection of a single antigen can elicit a potentially strain-transcending immune response. These RNA vaccines, while promising, still need to be evaluated in the field, however it does provide evidence of the potential for the mRNA platform to be employed against parasitic diseases as well.

#### 4.1.2 Immunotherapies for cancers prevalent in Africa

Cancer, a relatively neglected but common disease in Africa, is likely to overtake infectious diseases as the leading cause of mortality (174). The prevalence and fatalities associated with the different types of cancers differ between the African regions and is likely linked to differences in lifestyle, genetics, socioeconomic factors, and infectious diseases. East, Central and West Africa bear the burden of cancers caused by infectious diseases. The most common is cervical cancer, caused by Human Papilloma virus (175). This is followed by hepatocellular carcinomas caused by hepatitis C virus (HCV) or HBV. There are prophylactic vaccines available for these diseases and cervical cancers can be cured if detected early enough. Unfortunately, the lack of vaccination programs and access to screening continues to contribute to rising new infections and mortalities. Chronic HBV infections are not curable which leaves a large proportion of the 82.3 million infected individuals at risk for the development of cancer (176). Thus, there is a need for curative therapeutics and therapeutic vaccines against chronic infections which can easily be developed with mRNA technologies.

Cancers linked to lifestyle or genetic factors such as breast, prostate, lung and colon cancers are more prevalent in Northern and Southern Africa (174). Individuals of African ancestry are also more susceptible to the development of these types of cancers (177). As mentioned previously, there are several mRNA-based cancer immunotherapies currently in clinical trials, however, most African countries are not currently equipped with the means to provide such therapies. In addition, the process of producing dendritic cell-based vaccines or CAR T cells, and identifying personalized neoantigens is too lengthy and costly, even for developed countries. Insufficient African genome-wide association studies [reviewed in (151)] and cancer studies focusing on genomics of cancers overrepresented in African populations (reviewed in (178)), as well as substantial genetic diversity (179, 180) hinders the development of suitable and personalized neoantigen cancer therapies for patients. These shortcomings thus limit the potential of mRNA-based cancer immunotherapies in Africa. More widely applicable immunotherapies, such as mRNA vaccines targeting well-characterized tumor specific antigens, mRNA-encoded immune stimulants, or monoclonal checkpoint inhibitors to reverse the immunosuppressive tumor environment, would be easier to implement in the interim [reviewed in (181)].

### 4.2 Improving storage stability at accessible temperatures

Current storage methods of mRNA-LNP drugs are also a concern, especially for mRNA vaccines, where cold-chain transportation is required. While conventional vaccines can commonly be stored at 4-8°C (182), the currently licensed COVID-19 mRNA vaccines require storage temperatures of -20°C and between -80°C and -60°C for Spikevax (Moderna) and Comirnaty (Pfizer-BioNTech) respectively (183). These ultra-low temperature storage requirements impact global distribution, particularly in low- and middle-income countries where appropriate infrastructure to store these vaccines, particularly during transport, may be lacking. Until recently, the storage of LNP-mRNA gene therapies had not been a major research focus. However, the recent limitations regarding access to the approved COVID-19 mRNA vaccines have emphasized the importance of optimizing the storage of mRNA therapies and vaccines at higher temperatures while maintaining physical stability and efficacy.

Incorrect storage of mRNA can result in chemical instability by processes such as oxidation and hydrolysis, resulting changes to the physical characteristics of the drug product which may alter functionality (reviewed in (184)). For example, hydrolysis of the phosphodiester bond may occur by deprotonation of the 3′-hydroxyl (OH) group of the ribose sugar, or acid catalyzed reversable isomerization (184, 185). Stability is highly dependent on factors such as pH, buffer composition and concentration, the presence of metal cations (reviewed in (184)), as well as the composition and physiochemical properties of the non-viral vector formulation (183). One study suggests that based on theoretical evidence, LNP-mRNA interactions may increase susceptibility of mRNA to degradation during storage (186).

Long-term storage of LNP-mRNA drugs and vaccines currently involves the use of a cryoprotectant to prevent aggregation. Non-permeable cryoprotectants such as sucrose and trehalose are used to allow vitrification of the surrounding aqueous solution (187). The Spikevax and Comirnaty vaccines both make use of sucrose at 87 mg/ml (188) and 20 mg/ml (189) respectively. Lyophilization, also known as freeze drying, is a common alternative storage method which has recently been interrogated for mRNA-based vaccines and could aid in promoting long term stability at higher temperatures. It involves removing the water from the mRNA-LNPs, by sublimation at low temperatures, forming a translucent “cake” which can then be reconstituted in nuclease-free water, or the desired buffer (190). Zhao et al. studied the long-term storage of mRNA encapsulated in ionizable lipid-like LNPs (LLNs). While 5% sucrose and trehalose were sufficient for freeze thawed and liquid nitrogen stored mRNA-LLNs to maintain size and mRNA expression, a minimum of 20% sugar was required to have similar results for lyophilization. When evaluated *in vivo*, mRNA-LLNs stored with 5% sucrose or trehalose in liquid nitrogen for 1 week and three months expressed luciferase similar to fresh LNPs. However, lyophilized mRNA-LLNs expressed significantly lower after just 1 week’s storage (191). In the absence of a cryoprotectant during lyophilization, Ball et al. showed that resuspension of siRNA-Lipidoid Nanoparticle lyophilized cakes required a minimum of 30% ethanol to maintain silencing ability, encapsulation efficiency, and LNP size (192). Higher percentages of sucrose or trehalose present in lyophilized samples maintained all parameters after resuspension of the RNA-LNP cake in de-ionized water alone. Buffer pH showed no difference in siRNA potency after storage at different temperatures in the absence of a cryoprotectant. However, pH and buffer composition or concentration was not examined for lyophilization, which could have an impact on the stability and integrity of the RNA and LNPs as well as their interactions with each other (i.e. encapsulation efficiency) (182).

Although these studies measure activity, physical characteristics, encapsulation efficiency of the RNA-LNPs, RNA integrity, lipid composition, and reconstitution of these LNPs at different time points and temperatures remains unknown. Ai et al. recently studied the physical LNP characteristics, encapsulation efficiency, and size of lyophilized mRNA-LNPs at different temperatures and time points, albeit only for 18 days (193). Lyophilized mRNA-LNPs stored at 4°C and 25°C were stable for at least 18 days, showing no changes in size, encapsulation, and RNA integrity, while those stored at 40°C displayed an increase in size, without loss of RNA integrity or encapsulation efficiency. *In vivo* results showed comparable luciferase expression and anti-SARS-CoV-2 IgG titers when compared to fresh mRNA-LNPs. In addition neutralization antibodies against different SARS-CoV-2 variants were established and transgenic mice were protected in an infection-based model (193). An in-depth investigation into lyophilization of mRNA-LNPs recently revealed that storage at 4°C and below allowed maintenance of LNP size, polydispersity index and encapsulation efficiency for 24 weeks (182). Room temperature (RT) and below were able to maintain RNA concentration for at least 24 weeks, however RNA integrity was more sensitive, with only storage at -80 and -20°C being sufficient to prevent degradation, while lipid stability and ratio was maintained regardless of storage temperature. Despite this reduction in RNA integrity, *in vivo* luciferase expression was maintained for storage at RT and below for up to 4 weeks, and 4°C and below for up to 24 weeks. When assessed more practically for an influenza vaccine showed no difference in antibody titers between the frozen mRNA-LNPs (traditional storage) and RT and 4°C lyophilized mRNA-LNPs stored for 12 and 24 weeks respectively (182). Zhang et al. state that their dendrimer-based LNPs are stable at 5°C, however this was solely based off size, and measurement of other characteristics (such as RNA integrity) and protein expression is still required to confirm this (90).

These studies provide the groundwork for future storage of mRNA vaccines and therapies at temperatures similar to that of current conventional vaccines. However, new methods of freeze drying require further optimization as the conventional batch drying process is very time consuming (182) which could slow down vaccine production times. Recently, spin freeze drying has emerged as an alternative technology which could significantly reduce the total drying time (194). This process involves freezing the product by spinning the vial rapidly on its longitudinal axis while freezing using an inert gas (195). This is followed by a drying step using an infrared heater. Spinning results in a thinner layer with an increased surface area, resulting in improved sublimation. The processing also allows for continuous monitoring of temperature (196) and different vials and batches are subjected to the exact same conditions, improving reproducibility and scalability (196–198).

While lyophilization is still in development, a temporary ultra-cold chain storage device known as Cryo-Vacc has been developed by a South African company, Renergen (199). This small, helium powered device has an adjustable temperature control range of between -70°C and -150°C, allowing it to transport and store vaccines (a minimum of 100 doses) that require ultra-low temperature storage for up to 30 days. However, this is not suitable for long-term storage of mRNA vaccines, therefore, the infrastructure to develop lyophilized mRNA-LNP vaccines at large scale may still be a necessity for Africa.

### 4.3 Establishing African mRNA vaccine manufacturing

**Figure 5:**
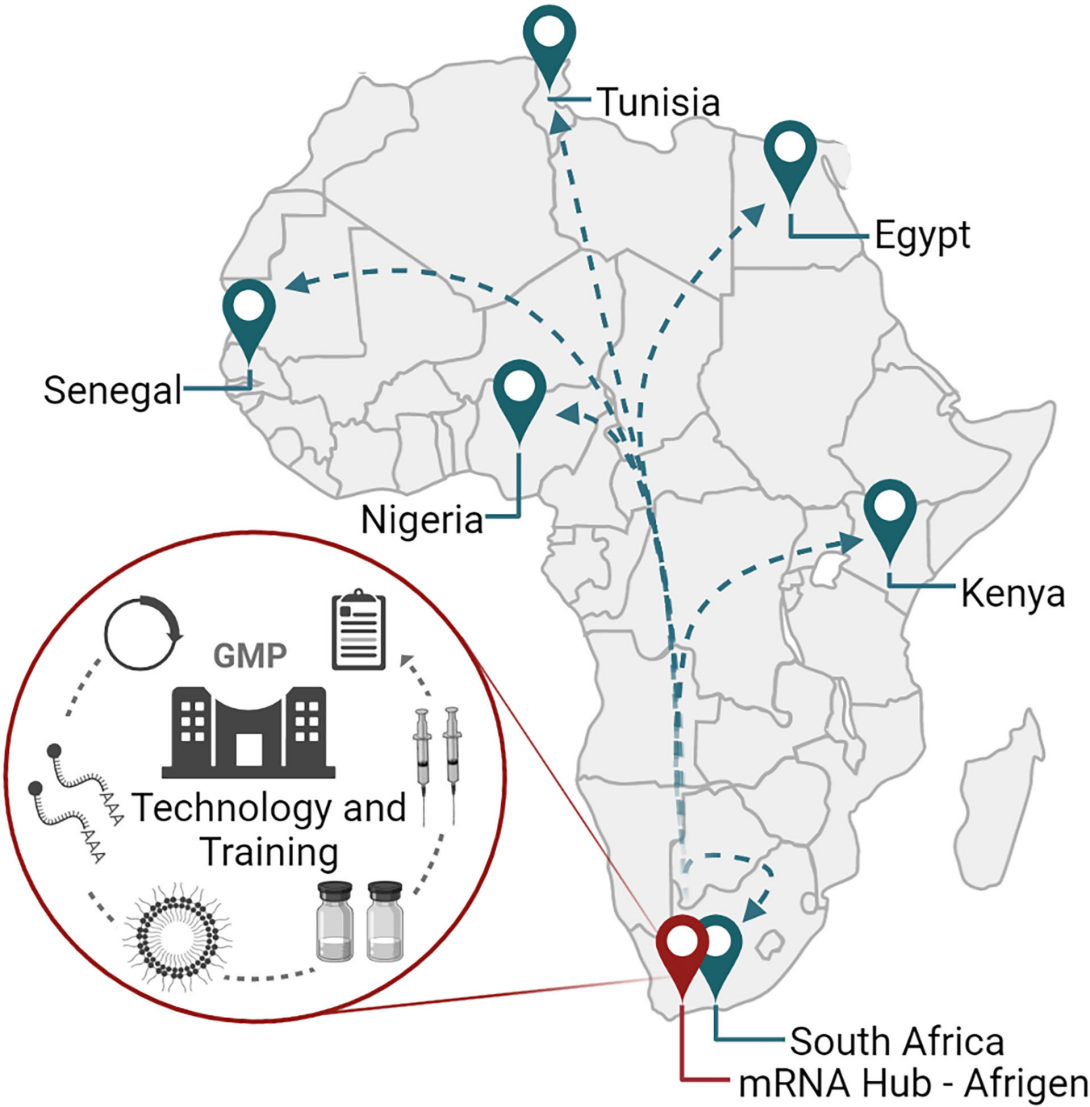
The mRNA vaccine technology hub and spokes program in Africa. The training and technology hub is located at Afrigen in Cape Town, South Africa. Afrigen is establishing mRNA vaccine production technologies at industrial scale for transfer to various spokes in low- and middle-income countries. To date, six African spokes have been identified: Egypt, Kenya, Nigeria, Senegal, South Africa, and Tunisia. Created using BioRender.com.

While the COVID-19 pandemic revealed how international cooperation between researchers, biotech and pharmaceutical companies, governments, philanthropies, investors, and non-governmental organizations could drive vaccine and therapeutic development, it was also a stark reminder of global vaccine inequalities observed in lower middle- and low-income countries (200). For Africa, equitable access to vaccines has been a longstanding challenge as infectious disease burdens for vaccine-preventable diseases alone remain high (201). The onset of the COVID-19 pandemic highlighted Africa’s dependence on global partners for vaccines and therapies, and a lack of local infrastructure for pandemic/epidemic preparedness. To establish local vaccine manufacturing capabilities in lower middle- and low-income countries, the WHO in association with the Medicines Patent Pool (MPP) and the Act-Accelerator/COVAX have launched the mRNA technology transfer program (202–204). The main objective of the program is to establish local COVID-19 mRNA vaccine manufacturing capacity, with Egypt, Kenya, Nigeria, Senegal, South Africa, and Tunisia listed as the first recipients of the technology in Africa (**Figure 5**). While the initial focus is on the production of COVID-19 vaccines, the mRNA platform is versatile and could be applied to different vaccines and therapies in the future.

Benefits of well-functioning African mRNA vaccine manufacturing facilities are clear. However, successfully implementing the program requires that several technical and socio-political challenges be met. Some of the important considerations include: 1) Materials required for manufacture of mRNA are rarely produced locally, and African countries are typically reliant on import of reagents such as enzymes and modified nucleotides. This adds expense and logistical complexity to the manufacturing pipeline; 2) Upskilling to increase human capacity with expertise in fields related to mRNA platforms is required to build vaccine manufacturing capabilities in the continent; 3) African countries are largely reliant on intellectual property that is generated in the so-called global north, which restricts freedom to operate (205). A priority of the WHO-sponsored African mRNA vaccination hub alongside the MPP, is generation of new IP that enables unencumbered vaccine manufacture; 4) Facilities for accredited manufacture of mRNA are limited and establishing resources that are required for large scale production will be essential. One major benefit of establishing mRNA vaccine facilities compared to other technologies, is the relatively small manufacturing footprint. Attention to ensuring that internationally approved standards are met is necessary; 5) Overcoming vaccine skepticism, which is not unique to Africa, may prevent wide acceptance of new technologies. Although the abovementioned problems are real and pose a threat to success of the African mRNA vaccination hub, awareness of these issues enables rational planning to mitigate the risks. Another important positive factor is that there is considerable good will from high-income countries that have experience with mRNA vaccine manufacture. Assistance with training, financial support, technology transfer, meeting regulatory requirements has been impressive and has facilitated progress of the project.

Building local vaccine manufacturing capabilities and infrastructure will aid in reducing Africa’s dependence on international partners for access to life-saving vaccines, with the added incentive to create employment opportunities and upskill the local workforce. Being able to drive research and design of novel vaccines and therapies will also allow countries to focus on important health care issues specific to their region. mRNA technology holds an exciting potential if these shortcomings are acted upon. Therapies such as neoantigen vaccines, therapeutic protein replacement therapies, gene editing technologies for major African genetic disorders such as sickle cell disease (206), and vaccines against prominent infectious diseases such as HIV, TB and Malaria can be developed, creating new opportunities to improve population health and cascade to improve socio-economic factors. Versatility of the platform allows modifications to be made to the target, delivery system, production and distribution methods tailored to the geographical area and population. Although this is a long-term goal, current expertise in the field and collaborations have sparked the beginning of the implementation of this technology into African laboratories, industry, and medicine.

## Author contributions

DK and NS contributed equally to this review by writing and creating the figures. KB contributed to writing of the review. KB, AE, and PA reviewed and edited the manuscript. All authors contributed to the article and approved the submitted version.

## Funding

Financial support from the South African National Research Foundation (Unique Grant Numbers: 120383), The Poliomyelitis Research Foundation, the South African Medical Research Council (SAMRC) through its Division of Research Capacity Development under the Research Capacity Development Initiative from funding received from the South African National Treasury, and extramural unit funding from the SAMRC is gratefully acknowledged. The content and findings reported/illustrated are the sole deduction, view and responsibility of the researcher and do not reflect the official position and sentiments of the SAMRC.

## Conflict of interest

AE, PA and KB are contracting partners of Afrigen Biologics and Vaccines through the mRNA Hub.

The remaining authors declare that the research was conducted in the absence of any commercial or financial relationships that could be construed as a potential conflict of interest.

## Publisher’s note

All claims expressed in this article are solely those of the authors and do not necessarily represent those of their affiliated organizations, or those of the publisher, the editors and the reviewers. Any product that may be evaluated in this article, or claim that may be made by its manufacturer, is not guaranteed or endorsed by the publisher.
